# Transcriptional Regulation of the *Angptl8* Gene by Hepatocyte Nuclear Factor-1 in the Murine Liver

**DOI:** 10.1038/s41598-020-66570-0

**Published:** 2020-06-19

**Authors:** Takuya Watanabe, Atsushi Ozawa, Shinnosuke Masuda, Satoshi Yoshino, Emi Ishida, Yuri Kondo, Shunichi Matsumoto, Akiko Katano-Toki, Kazuhiko Horiguchi, Yasuyo Nakajima, Eijiro Yamada, Takuya Tomaru, Tsugumichi Saito, Sumiyasu Ishii, Nobuyuki Shibusawa, Shuichi Okada, Tetsurou Satoh, Masanobu Yamada

**Affiliations:** Department of Internal Medicine, Division of Endocrinology and Metabolism, Gunma University Graduate School of Medicine, 3-39-15 Showa-machi, Maebashi, 371-8511 Japan

**Keywords:** Gene regulation, Molecular biology

## Abstract

Brief refeeding times (~60 min) enhanced hepatic Angptl8 expression in fasted mice. We cloned the mouse *Angptl8* promoter region to characterise this rapid refeeding-induced increase in hepatic Angptl8 expression. Deletion of the −309/−60 promoter region significantly attenuated basal promoter activity in hepatocytes. A computational motif search revealed a potential binding motif for hepatocyte nuclear factor 1α/1β (HNF-1α/β) at −84/−68 bp of the promoter. Mutation of the HNF-1 binding site significantly decreased the promoter activity in hepatocytes, and the promoter carrying the mutated HNF-1 site was not transactivated by co-transfection of HNF-1 in a non-hepatic cell line. Silencing Hnf-1 in hepatoma cells and mouse primary hepatocytes reduced Angptl8 protein levels. Electrophoretic mobility-shift assays confirmed direct binding of Hnf-1 to its *Angptl8* promoter binding motif. Hnf-1α expression levels increased after short-term refeeding, paralleling the enhanced *in vivo* expression of the Angptl8 protein. Chromatin immunoprecipitation (ChIP) confirmed the recruitment of endogenous Hnf-1 to the *Angptl8* promoter region. Insulin-treated primary hepatocytes showed increased expression of Angptl8 protein, but knockdown of Hnf-1 completely abolished this enhancement. HNF-1 appears to play essential roles in the rapid refeeding-induced increases in *Angptl8* expression. HNF-1α may therefore represent a primary medical target for ANGPTL8-related metabolic abnormalities. The study revealed the transcriptional regulation of the mouse hepatic *Angptl8* gene by HNF-1.

## Introduction

Angiopoietin-like proteins (Angptls) are a family of proteins structurally similar to angiopoietins. Previous reports on Angptls have identified them as key regulators of circulating triglyceride(TG) levels, implicating them as potential new drug targets for treatment of metabolic syndrome^1,2^ *ANGPTL8*, alternatively called the TD26, lipasin, C19orf80, or RIFL (refeeding induced fat and liver) gene, was originally identified as a novel adipocyte-enriched insulin target gene with a role in lipid metabolism^3–5^. (A previously reported identification of Angptl8 as ‘Betatrophin,’ a factor controlling the differentiation and proliferation of pancreatic β cells^6^, retracted article), was later determined to be incorrect^7–10^. Ren *et al*. found that the *RIFL* gene encodes a predicted protein of 22kD with homology to Angptl3^3^, Furthemore, the murine white and brown adipose tissue (WAT and BAT) and liver were highly enriched in the *RIFL* transcript, and the level increased ~80-fold in WAT and 12-fold in liver, following 8 h refeedings of fasting mice^3^. Several studies with Angptl8-deficient or Angptl8-overxpressing rodents have demonstrated that Angptl8, which is homologous to the N-terminal domain of Angptl3, modulates circulating TG clearance by inhibiting lipoprotein lipase (LPL) activity in the presence of Angptl3^3–5,7,8,11^. Recent human studies have shown that serum ANGPTL8 levels are increased in subjects with Type 1 diabetes (T1D)^12^, obesity^13^, Type 2 diabetes (T2D)^13–15^, and non-alcoholic fatty liver disease (NAFLD)^16^. Most recently, high levels of circulating ANGPTL8 were determined in patients with infections, where ANGPTL8 played a role in selective autophagy in the inflammatory responses by controlling the activation of nuclear factor-κB^17^.

In humans, ANGPTL8 is mainly expressed in the liver^5^; therefore, elucidating the mechanism that regulates the hepatic expression of ANGPTL8 is important for establishing the protein as a novel biomarker for metabolic diseases in human subjects. Several *in vitro* or *in vivo* studies have been conducted^3–5,7,8,18,19^, but the regulatory mechanisms controlling *Angptl8* gene expression remain unclear. Previous reports have demonstrated that Angptl8 expression in mice is suppressed by fasting and dramatically induced by refeeding^4,5,11,19^. However, no reports to date have analysed the expression of Angptl8 after a short period of refeeding in fasted mice. Here, we show that the hepatic Angptl8 expression is significantly and rapidly increased (within 60 min) after the start of refeeding. We also show that HNF-1 is essential for Angptl8 expression in the liver.

## Results

### Refeeding increased Angptl8 expression at both the mRNA and protein levels in mouse liver

**Figure 1:**
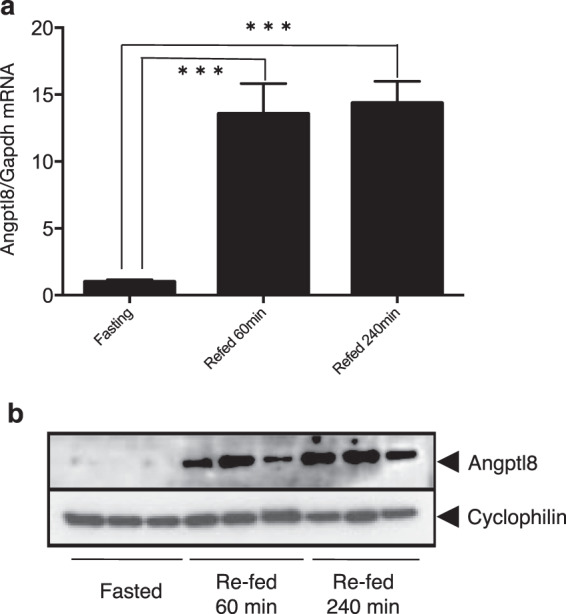
The expression of hepatic *Angptl8* mRNA and protein were increased by refeeding after fasting. (**a**) After a 12 h fast, 20-week-old male mice were re-fed *ad libitum* and euthanised after 60 and 240 min. Total RNA of the liver was isolated and subjected to qPCR to quantify Angptl8 mRNA levels. Data represent mean ± SEM from 5 mice at each time point from triplicate PCR samples. The experiment was repeated once with similar results. Asterisks indicate significant differences from fasting *Angptl8* expression levels (***p < 0.001). (**b**) Whole cell lysates of the liver were prepared from three different mice at each time point and subjected to immunoblotting using anti-Angptl8 and anti-cyclophilin antibodies. The Angptl8 and the cyclophilin blots were conducted by re-probing the same parts of the same membrane.

After a 12 h fast, 20-week-old male mice were re-fed *ad libitum* and euthanised after 60 and 240 min. All livers were harvested and Angptl8 mRNA levels were measured by qPCR (Fig. 1a). When compared with expression in the fasted state, hepatic Angptl8 mRNA expression was significantly increased by 13.6 ± 2.27 fold (p < 0.001) after 60 min and by 14.4 ± 1.63 (p < 0.001) fold after 240 min after refeeding. The expression levels of Angptl8 protein were also increased in the livers from three different mice after 60 and 240 min of refeeding (Fig. 1b). These findings suggested that the levels of *Angptl8* mRNA and protein in the murine liver were both rapidly increased after refeeding through rapid transcriptional activation of the *Angptl8* gene.

### The hepatocyte-specific transcription factor HNF-1 was required for hepatocyte-specific activation of the mouse Angptl8 promoter

**Figure 2:**
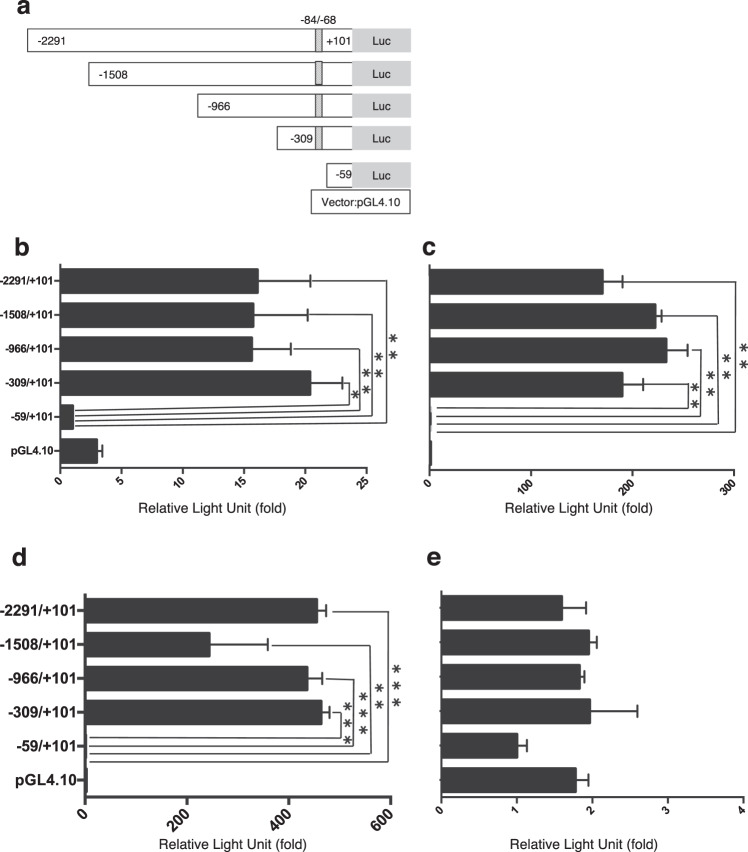
Promoter deletion analyses of mouse *Angptl8* gene in liver- or non-liver-derived cell lines and mouse primary hepatocytes. (**a**) Schematic representation of mouse *Angptl8* gene promoter constructs. The serially deleted promoter regions (−2291/+101, −1508/+101, −966/+101, −309/+101, and −59/+101) were ligated to the pGL4.10 luciferase reporter vector (Luc). The position of the consensus-binding motif for hepatocyte nuclear factor-1 (HNF-1) at −84/−68 (GGTTAACCATTGACCAG) is indicated as oblique boxes. These promoter constructs were transfected to mouse hepatoma-derived Hepa1–6 cells **(b)**, human hepatoma-derived HepG2 cells **(c)**, mouse primary hepatocytes **(d)** or HeLa cells originated from human uterine cervical cancer **(e)**. The luciferase activity of the −59/+101 construct was set as 1 and data represent mean ± SEM from three independent experiments with triplicate determinations. Asterisks indicate significant differences from the activity of −59/+101 construct (*p < 0.05, **p < 0.01 or ***p < 0.001).

We investigated the transcriptional regulation of *Angptl8* gene in the liver by first cloning various lengths of the promoter fragment of the *Angptl8* gene. As shown in Supplementary Fig. 1, we indicated the T residue, the first nucleotide of exon1, as +1 in this study. We first cloned the −2291/+101 promoter fragment of the gene by genomic PCR. This allowed construction of serially deleted promoter fragments (Fig. 2a) to identify the promoter regions that were critical for activation of *Angptl8* gene expression in the liver. These promoter fragments were then inserted into a luciferase reporter vector and transiently transfected into mouse hepatoma-derived Hepa1–6 cells, human hepatoma-derived HepG2 cells, mouse primary hepatocytes and HeLa cells originating from human uterine cervical cancer. As shown in Figs. 2b–d, the −2291/+101 fragment showed strong activity in hepatic cancer cell lines and in mouse primary hepatocytes. In both these cell types, similar promoter activities were detected for the −1508/+101, −966/+101 and −309/+101 constructs. Further deletion of the −309/−60 region completely abrogated the promoter activities. In contrast to hepatocytes, HeLa cells showed very weak promoter activities (Fig. 2e). These findings indicated that the promoter region between −309 and −60 contains an element critical for *Angptl8* gene expression in the liver.

**Figure 3:**
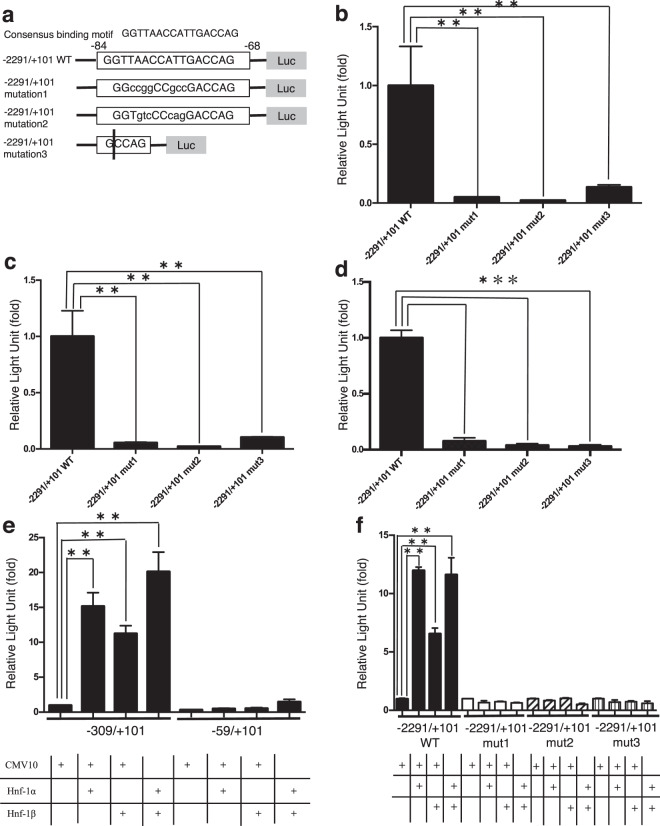
(**a–d**) Mutations of the HNF-1 binding site at −84/−68 abrogated the basal *Angptl8* gene promoter activities in hepatocytes. (**a**) Point mutations (indicated as lower case letters) or 12-base pair deletion in the HNF-1 binding site at −84/−68 were introduced into the −2291/+101 construct (see Materials and Methods) and transfected into Hepa1–6 cells **(b)**, HepG2 cells **(c)** or mouse primary hepatocytes **(d)**. Promoter activities are expressed as luciferase activities relative to those of the wildtype −2291/+101 construct, which was assigned a value of 1. Data represent mean ± SEM from three independent transfections with triplicate determinations. Asterisks indicate significant differences from the wildtype −2291/+101 activities (**p < 0.01 or ***p < 0.001). (**e**) Basal promoter activity of the mouse *Angptl8* gene requires co-transfected HNF-1 in non-hepatocytes. The −309/+101 reporter construct with the wildtype HNF-1 binding motif at −84/−68 or −59/+101 construct lacking the HNF-1 binding site was co-transfected with HNF-1α and/or β expression vector into HeLa cells. A CMV10 expression vector was co-transfected as a control. Promoter activities are expressed as luciferase activities relative to those of the −309/+101 construct in the absence of co-transfected HNF-1 expression vector, which was assigned a value of 1. Data represent mean ± SEM from three independent transcription with triplicate determinations. Asterisks indicate significant differences from −309/+101 reporter activities in the absence of co-transfected HNF-1 (**p < 0.01). (**f**) Basal promoter activity of the mouse *Angptl8* gene requires the HNF-1 binding site at −84/−68 in non-hepatocytes. The −2291/+101 reporter construct with the wildtype or differently mutated HNF-1 binding site (mut1, 2 and 3) at −84/−68 were co-transfected with HNF-1α and/or β expression vector into HeLa cells. Promoter activities are expressed as luciferase activities relative to those of the −2291/+101 construct in the absence of co-transfected HNF-1 expression vector, which was assigned a value of 1. Asterisks indicate significant differences from −2291/+101 activities in the absence of cotransfected HNF-1 (**p < 0.01).

We then performed a computer search using the TRANSFAC program (GeneXplain GmbH, Wolfenbuttel, Germany) to identify potential binding sites for transcription factors in the −309/−60 region. This search identified the presence of a consensus-binding motif for HNF-1 at −84/−68 (Fig. 3a and Supplementary Fig. 1). HNF-1 (HNF-1α and β) is a hepatocyte-specific transcription factor that binds to a sequence required for the hepatocyte-specific transcription of several genes. We further validated the importance of the Hnf-1 binding site at −84/−68 for *Angptl8* promoter activation in the liver by introducing two distinct series of mutations (mutation 1 and 2) or a 12-bp deletion (mutation 3) to the Hnf-1 binding site in the −2291/+101fragment (Fig. 3a). The use of these mutations was based on a previous report^20^ showing that these mutations had no ability to bind to HNF-1. The reporter genes harbouring these mutations were transfected in parallel with the wildtype promoter into Hepa1–6 and HepG2 cells and mouse primary hepatocytes. As shown in Figs. 3b–d, three mutations in the Hnf-1 binding site significantly reduced promoter activity in all three cell types.

We then determined whether overexpression of HNF-1 could activate the *Angptl8* gene promoter in HeLa cells. The −309/+101 reporter was cotransfected with or without human HNF-1α and/or HNF-1β expression vectors and *Angptl8* promoter activity was assessed with luciferase assays. As shown in Fig. 3e, cotransfection of HNF-1α and HNF-1β significantly and synergistically activated the promoter activity in non-hepatocytes. Moreover, this activation by cotransfected HNF-1α and HNF-1β was completely abolished in HeLa cells transfected with the −2291/+101 reporter carrying the mutations in the HNF-1 binding site at −84/−68 (mut1, mut2 and mut3) (Fig. 3f). These findings clearly indicated that the HNF-1 binding site at −84/−68 is essential for specific activation of *Angptl8* gene expression in the liver.

### HNF-1 was an essential factor required for the mouse Angptl8 protein expression in hepatocytes

**Figure 4:**
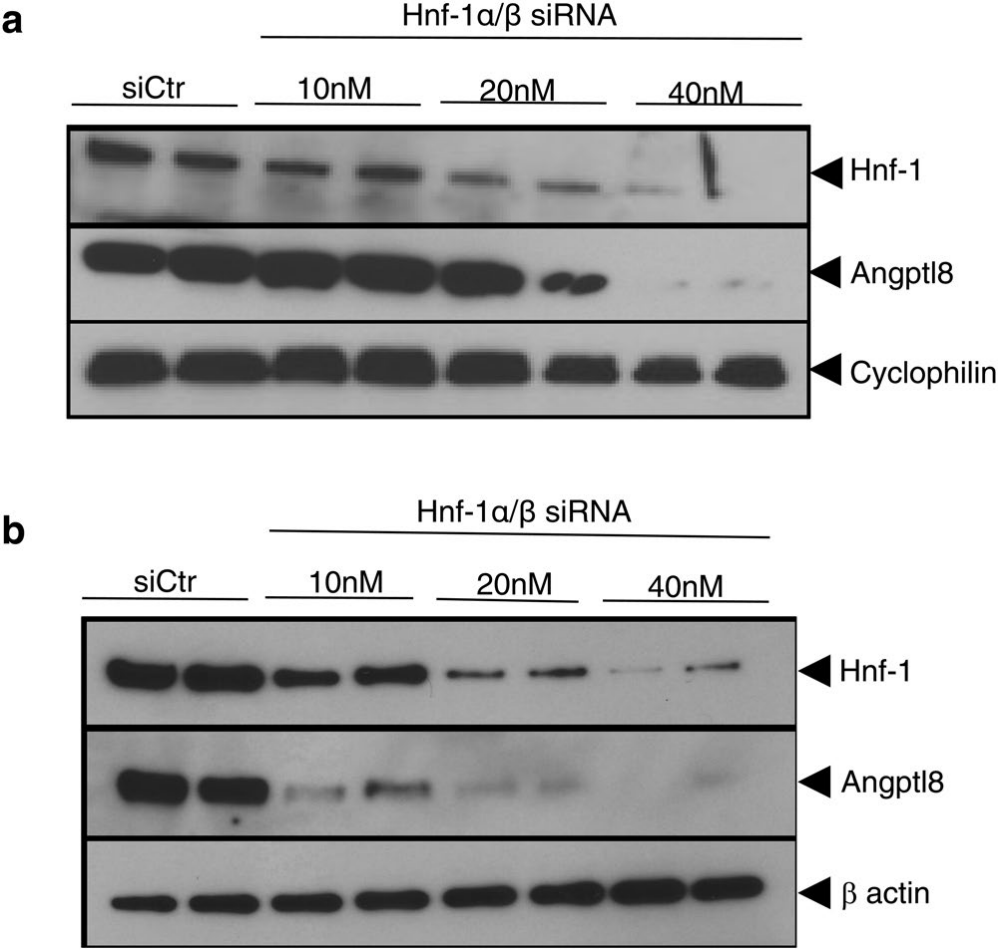
Knockdown of endogenous Hnf-1 reduced Angptl8 protein expression levels in Hepa1–6 cells (**a**) and mouse primary hepatocytes (**b**). At 24 h after transfection of both siHnf-1α and siHnf-1β or control siRNA (siCtr), whole cell lysates were prepared and subjected to SDS-PAGE. Immunoblotting was performed using anti-Angptl8, anti-cyclophilin and anti-β-actin antibodies. Increasing amounts of transfected siHnf-1α and siHnf-1β reduced Angptl8 protein levels in a dose-dependent manner in both Hepa1–6 cells and primary hepatocytes. The blot of Hnf-1 and the blot of Angptl8 were cropped from different parts of the same membrane. The blot of cyclophilin and β-actin was cropped from a different membrane but using the same whole cell lysates used for anti-Hnf-1 and anti-Angptl8 blotting.

We examined whether HNF-1 is critical to the hepatic expression of endogenous Angptl8 by transfection of Hepa 1–6 cells and mouse primary hepatocytes with siRNA targeting endogenous Hnf-1α and Hnf-1β or control siRNA. We then measured the Angptl8 protein levels by immunoblotting. As shown in Fig. 4a, siHnf-1α/β expression significantly and dose-dependently reduced Hnf-1 protein levels 24 h after siRNA transfection. Endogenous Angptl8 protein levels were also dose-dependently reduced by knockdown of Hnf-1α/β in Hepa 1–6 cells. Furthermore, the knockdown of Hnf-1α/β in the primary hepatocyte with 10 nM siRNA also clearly reduced the expression of endogenous Angptl8 (Fig. 4b). These findings suggest that Hnf-1 plays an important role in the basal expression of endogenous Angptl8 in hepatocytes. Exogenous expression of HNF-1 in non-hepatic cells (HeLa cells) that do not express endogenous HNF-1 did not induce the expression of the Angptl8 protein (Supplementary Fig. 2). This could because HeLa cells lack endogenous Angptl8 expression (Supplementary Fig. 2, left panel).

### Direct binding of Hnf-1 to the region −84 and −68 bp in the Angptl8 gene

**Figure 5:**
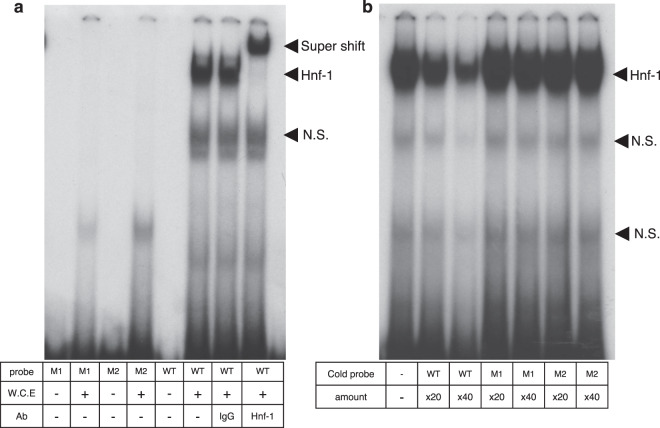
Binding of Hnf-1 to the −84/−68 region of the Angptl8 gene. (**a**) Electrophoretic mobility-shift assays (EMSA) showed that Hnf-1 in whole cell extracts from Hepa1–6 cells significantly bound to the wildtype probe of the Angptl8 promoter lesion –94/−60 that included the Hnf-1 binding consensus motif. This complex was supershifted with a specific antibody against Hnf-1. By contrast, no protein binding was observed using mutant probes (M1 or M2) that contain nucleotide mutations in the Hnf-1 binding consensus motif. (**b**) The amount of Hnf-1 bound to the region –94/−60 was reduced in a dose-dependent manner by adding 20- or 40-fold molar excess cold (non-^32^P labelled) probes. Adding non-labelled probes including mutated nucleotides in the Hnf-1 binding consensus motif did not alter the amount of Hnf-1 binding. W.C.E indicates the whole cell extract and N.S indicates the non-specific binding.

We investigated whether Hnf-1 directly binds to the −84 and −68 bp region responsible for the promoter activity of the Angptl8 gene by performing electrophoretic mobility-shift assays (EMSA). We used radiolabelled fragments containing region 5′-AACCATGGCCGGTTAACCATTGACCAGGGGGGTCA-3′ as a wild type (WT) probe, and mutant probes that included nucleotide changes (M1: 5′- AACCATGGCCGGCCGGCCGCCGACCCAGGGGGGGTCA -3′ and M2: 5′-AACCATGGCCGGTGTCCCGAGGACCAGGGGGGGGTCA -3′). As shown in Fig. 5a, the components from whole cell extracts of Hepa1–6 cells were bound to the WT probe but not to the M1 or M2 probes. The addition of an antibody against Hnf-1 induced a clear super-shift the band, confirming that the binding was a complex containing Hnf-1. The addition of unlabelled oligonucleotides containing the wildtype Hnf-1 binding motif interfered with the binding of the complex containing Hnf-1 to the WT probe (Fig. 5b). By contrast, the addition of unlabelled oligonucleotides containing the mutant binding motif did not interfere with the binding of the complex to the WT probe. These findings indicated that the endogenous Hnf-1 in Hepa1–6 cells was bound directly to the promoter −84/−68 region of the mouse Angptl8 gene.

### Refeeding increased the expression of the Hnf-1α gene at both the mRNA and protein levels in the mouse liver but did not affect Hnf-1β gene expression

**Figure 6:**
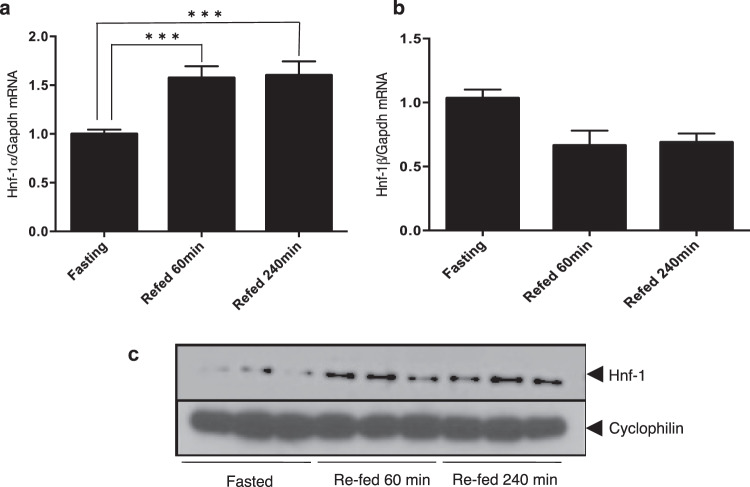
(**a,b**) The mRNA levels of Hnf-1α, but not Hnf-1β, in the liver were significantly increased after refeeding. After a 12 h fast, mice were refed *ad libitum* and sacrificed after 60 and 240 min. Total RNA of the liver was isolated and subjected to qPCR to quantify mRNA levels of Hnf-1α or β. Data represent mean ± SEM from 5 mice at each time point from triplicate PCR samples and the experiment was repeated once with similar result. Asterisks indicate the significant difference from mRNA levels in the liver of fasted mice (***p < 0.001). (**c**) Hepatic Hnf-1 protein levels were increased by refeeding. After a 12 h fast, mice were refed *ad libitum* and euthanised after 60 and 240 min. Whole cell lysates of the liver were prepared from three different mice at each time point and subjected to immunoblotting using anti-Hnf-1 and anti-cyclophilin antibodies. The blot of Hnf-1 and the blot of cyclophilin were cropped from different parts of the same membrane.

We also examined the potential involvement of Hnf-1 in the rapid *in vivo* upregulation of Angptl8 induced by refeeding. We measured the Hnf-1 mRNA and protein levels in mouse livers after 60 and 240 min of refeeding and after a 12 h fast. The expression of Hnf-1α mRNA, determined by qPCR, was increased after 60 min (1.6 ± 0.12 fold; p < 0.001) and 240 min (1.6 ± 0.14 fold; p < 0.001) of refeeding (Fig. 6a). By contrast, the expression of Hnf-1β mRNA was not affected by refeeding (Fig. 6b, no significant differences were noted for any comparison of the three groups). In parallel with the rapid increase of Angptl8 protein levels after refeeding (Fig. 1b), we observed a similar increase in the levels of hepatic Hnf-1 protein after 60 and 240 min of refeeding (Fig. 6c). This result suggested that the protein recognised by the anti-Hnf-1 antibody in the immunoblots reflected increases in the levels of Hnf-1α mRNA but not Hnf-1β mRNA. These findings suggested that the levels of Hnf-1α, but not Hnf-1β was increased in the mouse liver by refeeding.

### Insulin increased the expression of both mRNA and protein of Hnf-1α in mouse hepatocytes

**Figure 7:**
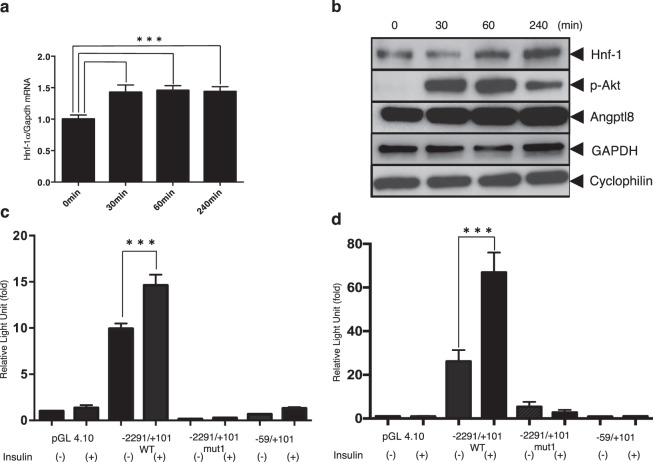
Insulin increased the expression of Hnf-1 mRNA and protein in Hepa1–6 cells (**a,b**). After 12 h from cell splits, Hepa1–6 cells were incubated with serum-free medium for 10 h and then transferred to a medium including 10 nM insulin. The cells were then collected at the indicated time points. (**a**) Total RNA isolated at each time point was subjected to qPCR to quantify Hnf-1α mRNA levels. Data represent mean ± SEM from triplicate PCR samples and the experiment was repeated once with similar results. Asterisks indicate a significant difference in the mRNA levels in Hepa1–6 cells at 0 min (***p < 0.001). (**b**) Whole cell lysates were prepared and subjected to immunoblotting using anti-Hnf-1, anti-p-Akt and anti-cyclophilin antibodies. The p-AKT was phosphorylated Akt. The blot of Hnf-1, the blot of p-AKT and the blot of cyclophilin were cropped from different parts of the same membrane. The blot of Angptl8 and cyclophilin was cropped from different membrane using the same whole cell lysates used for anti-Hnf-1, p-AKT and cyclophilin blotting. (**c,d**) Incubation with 10 nM insulin stimulated the promoter activity of Angptl8, including consensus binding motif for Hnf-1, in Hepa1–6 cells (**c**) and mouse primary hepatocytes (**d**). The −2291/+101 reporter construct with the wildtype Hnf-1 binding motif, −2291/+101 construct with the mutated Hnf-1 binding motif, or −59/+101 construct lacking the Hnf-1 binding site were transfected into Hepa1–6 cells or mouse primary hepatocytes. Ten hours after transfection, the cells were incubated with serum-free medium for 4 h. The cells were then incubated 2 h with or without 10 nM insulin. Promoter activities are expressed as luciferase activities relative to those of the pGL 4.10 construct in the absence of insulin stimulation, which was assigned a value of 1. Data represent mean ± SEM from three independent transcription with triplicate determinants. Asterisks indicate the significant difference in the −2291/+101 WT construct with insulin stimulation from the −2291/+101 WT reporter activities in the absence of insulin stimulation (***p < 0.001).

Several previous studies have investigated Angptl8 as an insulin target gene^3–5^. Our findings indicated that even a relatively short refeeding increased the *in vivo* expression of Hnf-1α; therefore, we tested whether insulin might stimulate the *in vitro* expression of Hnf-1α. As shown in Fig. 7a, a 30 min stimulation with 10 nM insulin caused a rapid increase in the expression of Hnf-1α mRNA in hepatocyte cells. The protein level of Hnf-1 was also increased 60 min after the increase in mRNA (Fig. 7b).

### Hnf-1 is required for enhancement of Angptl8 expression by insulin

We also investigated whether insulin treatment in Hepa 1–6 cells and mouse primary hepatocytes could activate the *Angptl8* gene promoter. After transfection with the −2291/+101 WT reporter, −2291/+101 mut1 reporter or the −59/+101 reporter, the cells were treated with 10 nM insulin. Luciferase assays (Fig. 7c,d) confirmed that stimulation of hepatocytes with 10 nM insulin significantly activated the promoter activity of the −2291/+101 WT reporter when compared with unstimulated control cells (p < 0.001). This activation by insulin was completely absent in the cells transfected with the −2291/+101 reporter carrying the mutations in the Hnf-1 binding site and in cells with the −59/+101 reporter lacking the Hnf-1 binding motif. These findings indicated an involvement of the HNF-1 binding site at −84/−68 in the insulin-dependent activation of *Angptl8* gene expression in hepatocytes. These data also further confirmed demonstrated that promoter activity required the HNF-1 binding site even in the presence of insulin.

**Figure 8:**
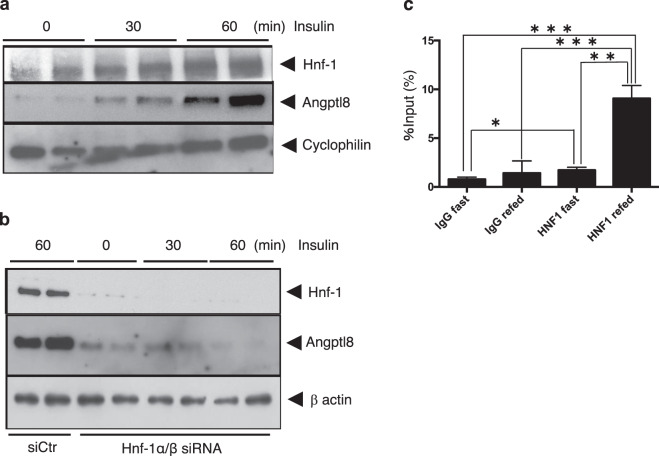
Insulin increased the protein expression level of Angptl8 in primary hepatocytes and required Hnf-1 (**a,b**). At 12 h after cell splits, mouse primary hepatocyte cells were incubated with serum-free medium for 2 h and then switched to a medium with 10 nM of insulin and cells were collected at the indicated time points. Whole cell lysates were prepared and subjected to immunoblotting using anti-Hnf-1, anti-Angptl8 and anti-cyclophilin antibodies. The blot of Hnf-1 and cyclophilin were cropped from different parts of the same membrane. The blot of Angptl8 was cropped from different membrane using the same whole cell lysates used for anti-Hnf-1and cyclophilin blotting (**a**). The same experiment was performed after Hnf-1 knockdown in mouse primary hepatocytes (**b**) using anti-β actin antibodies instead of anti-cyclophilin antibodies. At 24 h after transfection of both 40 nM siHnf-1α and siHnf-1β or control siRNA (siCtr), the cells were stimulated by incubation in medium containing 10 nM insulin. Whole cell lysates were prepared and subjected to SDS-PAGE. SiCtr samples were collected after 60 min from insulin administration. In the absence of Hnf-1, insulin did not increase the expression level of Angptl8 protein. Hnf-1 recruitment to the Hnf-1 binding site in Angptl8 promoter could be enhanced by insulin in *in-vivo* ChIP assays (**c**). After a 12 h fast, the mice were sacrificed or re-fed *ad libitum* and euthanised after 60 min. Liver homogenates from each mouse were immunoprecipitated with anti-HNF-1 antibody or pre-immune IgG (IgG). A ChIP assay was carried out as described in the Materials and Methods section. The experiments were performed three times, and each PCR was performed in triplicate. The results of the separate experiments were combined and represent mean ± SEM. An asterisk indicates a significant difference (*p < 0.05, **p < 0.01 or ***p < 0.001).

To confirm our findings and establish a pathophysiologic significance, we determined whether insulin stimulation could increase the protein expression of Angptl8 in mouse hepatocytes and whether the process requires HNF-1. Stimulation of primary hepatocytes with 10 nM insulin for 30 min increased the expression of both Hnf-1 and Angptl8 protein (Fig. 8a). However, Hnf-1α/β knockdown cells showed no increase in Angptl8 protein expression in response to insulin (Fig. 8b). Attenuation of Hnf-1 expression by 40 nM siRNA decreased the expression of endogenous Angptl8 and expression was not restored by addition of insulin. These data indicated a requirement for Hnf-1 for the enhancement of Angptl8 expression by insulin.

We also investigated the possible recruitment of endogenous Hnf-1 protein to the consensus binding motif in the −84/−68 bp legion of the mouse Angptl8 gene promoter. We did this by conducting chromatin immunoprecipitate (ChIP) assays in Hepa1–6 cells and in mouse liver tissue from fasted or re-fed mice using a set of primers that amplified the −170 to +102 bp region. As shown in Supplementary Fig. 3, in the absence of insulin stimulation, no significant recruitment of endogenous Hnf-1 was observed into the fragment containing the Hnf-1 binding site at −84/−68 (IgG Insulin(−) vs Hnf-1 Insulin(−)). However, stimulation with insulin significantly increased the recruitment of Hnf-1 into this region (IgG Insulin(+) vs Hnf-1 Insulin(+); p < 0.001). In the re-fed mice, Hnf-1 in liver tissues was recruited more significantly into the Angptl8 promoter fragment containing the Hnf-1 binding site at −84/−68 (Fig. 8c). These ChIP assay data suggested that the endogenous Hnf-1 protein could be recruited to the consensus binding motif in the −84/−68 bp region of the mouse Angptl8 gene promoter.

## Discussion

Angptl8 mRNA is highly expressed in mouse liver and brown fat, and moderately in subcutaneous fat, perigonadal fat, the kidneys, small intestine and heart^4,5^. In humans, ANGPTL8 mRNA is almost exclusively expressed in the liver, with limited expression in fat, brain, rectum and heart tissues^3–5^. Previous reports have shown an induction of Angptl8 mRNA in the liver, BAT, and WAT tissue in mice fed a high-fat diet, whereas Angptl8 expression was suppressed in fasting mice^4,5,11^. Refeeding following a period of food deprivation also enhanced the mRNA expression of Angptl8 within 8–12 h^3,4^. Dang *et al*. showed a relationship between the expression of Angptl8 and fasting/feeding signals over 4 h periods^19^. They found that the liver X receptor α (LXRα) upregulates hepatic Angptl8 expression through four LXR response element (LXREs) within the promoter region of the gene and that glucocorticoids negatively regulate Angptl8 expression during fasting^19^. The LXRs (α/β) play critical roles in the transcriptional regulation of lipid metabolism, and activation of LXRs increases the synthesis of hepatic fatty acids and the secretion of very-low-density lipoprotein (VLDL), thereby resulting in hypertriglyceridaemia^21^. Both Angptl3 and Angptl8 are primary targets of LXRs^22^.

Tseng *et al*. revealed that expression of human ANGPTL8 mRNA in HepG2 cells is induced by thyroid hormone^18^. This finding indicates that transcriptional regulation of the gene depends on the thyroid hormone receptor (TR), which directly binds to the upstream region of the human ANGPTL8 gene promoter. Previous reports have demonstrated that thyroid hormone directly regulates or crosstalks with nutrient-activated nuclear receptors to regulate lipid-associated gene transcription associated with hepatic lipid homeostasis^23–26^. In the present study, we demonstrated that even relatively short-term refeeding of fasted mice increases the hepatic expression of Angptl8 at both the mRNA and protein levels, suggesting that refeeding induces a rapid transcriptional activation of the *Angptl8* gene (Fig. 1a,b). Subsequent experiments confirmed a strict dependence of transcriptional regulation of the mouse hepatic *Angptl8* gene on the binding of HNF-1α and/or HNF-1β to the HNF-1 consensus-binding motif located in −84/−68 in the promoter region (Fig. 2a and Supplementary Fig. 1).

HNF-1 consists of two isoforms, HNF-1α^27^ and HNF-1β^28^. These two isoforms form homodimers or heterodimers that then activate the transcription of target genes by directly binding to the gene promoters^29,30^. These role of these transcription factors was originally identified as maintenance of expression of hepatic genes, such as *albumin, a1-antitrypsin and α- and β-fibrinogen*, and they were considered to be essential regulators of bile acid and plasma cholesterol metabolism^31^. Knockdown of the *TCF1* gene that encodes the three HNF-1α polypeptides confirmed an important role of this gene in the control of glucose homeostasis in mice^32–34^. Inactivation of the gene encoding HNF-1β has confirmed its essential role in the differentiation of visceral endoderm and in the genetic network th at directs the expression of HNF-4^35,36^. Mutations in the genes encoding HNF-4, HNF-1α and HNF-1β induce hyperglycaemia and are responsible for the type 1, type 3 and type 5 forms, respectively, of maturity-onset diabetes of the young (MODY)^37–40^. In the present study, non-liver derived HeLa cells showed no promoter activity, in all likelihood because of the lack of endogenous expression of HNF-1 (Fig. 2e). Co-transfection with HNF-1α and/or HNF-1β led to strong promoter activity of the *Angptl8* gene in non-liver derived cells (Fig. 3e). However, exogenous expression of HNF-1 failed to promote the expression of the Angptl8 protein in non-hepatic cells. This may because HeLa cells lack endogenous Angptl8 expression (Supplementary Fig. 2).

Dan *et al*. elegantly showed the mechanism of transcriptional regulation of the Angptl8 gene by LXRα and glucocorticoids through a possible binding motif in the promoter legion (−1980/−1971, −1943/−1934)^19^; however, our series of promoter deletions (−2291/+101 ~ −309/+101) showed a high promoter activity in liver-derived cell lines and mouse primary hepatocytes (Fig. 2b–d). These data revealed that the HNF-1 level could determine the basal promoter activity and the expression of the mouse *Angptl8* gene through the binding motif in the 5′ UTR of the murine liver gene. Our subsequent series of experiments also demonstrated that Hnf-1 was recruited to the binding motif (Figs. 5 and 8c and Supplementary Fig. 3), and that mutations inside the binding motif dramatically decreased the transcriptional activity of the gene (Figs. 3a–d,f). By binding to the HNF-1 binding motif in the *Angptl8* gene promoter region, both HNF-1α and HNF-1β could activate the promoter activity in these cell lines (Fig. 3e,f). In fact, the knockdown of both Hnf-1α and Hnf-1β decreased the expression of Angptl8 protein in both mouse hepatoma cells and mouse primary hepatocytes (Fig. 4a,b). A more interesting finding, however, is that the expression of the Angptl8 protein at various time points of fasting and refeeding almost paralleled the expression level of Hnf-1α, but not Hnf-1β (Fig. 1 and 6). These data demonstrate that HNF-1β has the potential to activate the *Angptl8* gene, but HNF-1α is the key regulator for *in vivo* activation of this gene. We also found increased amounts of Hnf-1α protein after relatively short refeeding periods (~60 min) (Fig. 6c).

The insulin response observed in cells is also interesting. Insulin regulates many cellular processes and the expression of more than 100 genes^41^. It controls gene transcription by modifying the binding of transcription factors on insulin-response elements or by regulating their transcriptional activities^41^. Several liver-specific genes are regulated by insulin, and many involve lipid synthesis molecules (e.g. fatty acid synthase, acetyl CoA carboxylase and stearoyl CoA desaturase), whereas others code for enzymes implicated in either glucose breakdown or transport (GLUT family members). Insulin control of HNF family members has been reported previously in a few studies; for example, Wolfrum *et al*. showed that Hnf-3β/Foxa-2-dependent transcriptional regulation is controlled by extracellular signals induced by insulin^42^. Similarly, two other reports showed that HNF-4 is a downstream target factor of the insulin signal^43,44^. Expression of Angptl8 is also responsive to insulin^3–5^, and we found that insulin stimulation increased the Hnf-1α expression at both the mRNA and protein levels in a relatively a short time (~60 min) (Fig. 7a,b). Insulin also stimulated the promoter activity of Angptl8, including the consensus binding motif for Hnf-1, in hepatoma cell lines and mouse primary hepatocytes (Fig. 7c,d), thereby confirming that the mechanism of insulin stimulation involves Hnf-1 binding.

The recruitment of Hnf-1 to the target region within the Angptl8 gene promoter was also verified by ChIP and *in-vivo* ChIP assays (Supplementary Fig. 3 and Fig. 8c). We also confirmed the involvement and pathologic importance of Hnf-1 in the insulin enhancement of Angptl8 expression in mouse primary hepatocytes (Fig. 8a,b). These results clearly demonstrated that basal and increased expression level of Angptl8 in liver requires Hnf-1, even in the presence of insulin, as the transcriptional activation of Angptl8 was suppressed in the absence of Hnf-1.

Taken together, the findings of the current study show that the hepatic expression of Angptl8 undergoes rapid transcriptional regulation during fasting/refeeding periods in mice. We have shown, for the first time, that the mouse *Angptl8* gene promoter is positively regulated by HNF-1, and we have identified an HNF-1 binding site in the mouse Angptl8 promoter. These findings will shed new light on the role of HNF-1 in lipid metabolism and suggest clinical applications for dyslipidaemia treatment.

## Materials and Methods

### Animals

Twenty-week-old male C57/BL6 mice were used in this study. The Institutional Animal Care and Use Committee of Gunma University Graduate School of Medicine (Maebashi, Gunma, Japan) approved all aspects of animal care and the experiments in any relevant details. We confirmed that all experiments were performed in accordance with relevant guidelines and regulations. Animals were maintained on a 12-h light, 12-h dark schedule (lights on at 0700 h) and fed standard laboratory chow and given water *ad libitum*. For fasting and re-feeding experiments, mice were fasted for 12 h (7:00 PM to 7:00 AM) during the dark cycle and re-fed for 60 and 240 minutes during the light cycle. Mice were euthanised after each refeeding time course. All livers were harvested and snap frozen in liquid nitrogen and stored at −80 °C. We state that

### RNA preparation and real-time quantitative PCR (qPCR)

Total RNA was extracted from mouse tissues and Hepa1–6 cells using ISOGEN (Nippon Gene, Tokyo, Japan). A 200 ng sample of total RNA was reverse transcribed with random hexamers using the Taqman reverse transcription reagent kit (Applied Biosystems Inc., Foster City, CA) according to the manufacturer’s protocol. Real-time qPCR assays were performed using an Applied Biosystems 7500 sequence detector (Applied Biosystems Inc., Foster City, CA) with the standard 40 cycles. Mouse Angptl8, Hnf-1α and Hnf-1β mRNA expressions were analysed using Taqman probes (Mm01175863_g1, Mm00493434_m1 and Mm00447459_m1, respectively; Applied Biosystems Inc., Foster City, CA). We confirmed that all PCR products were on the standard curve by parallel PCR conducted with serially diluted cDNA samples. The amplification was normalised using mouse glyceraldehyde-3-phosphate dehydrogenase (Gapdh) mRNA (Mm99999915_g1, Applied Biosystems Inc., Foster City, CA).

### Immunoblotting

Whole cell lysates prepared from mouse liver, Hepa1–6 cells and mouse primary hepatocytes, and 20 µg of protein were subjected to SDS-PAGE. The separated proteins were then immunoblotted as described previously using an anti-betatrophin (Angptl8) rabbit polyclonal antibody (no. 7619, ProSci, Poway, CA), an anti-HNF-1 antibody (sc-8986 ×, Santa Cruz Biotechnology, Inc., Santa Cruz, CA), an anti-Phospho-Akt (Ser473) (D9E) antibody (#4060, Cell Signaling Technology., Danvers, MA), an anti-GAPDH antibody (ab9485, abcam, Cambridge, UK), an anti-cyclophilin A antibody (Upstate., Lake Placid, NY) and an anti-β actin antibody (#4967, Cell Signaling Technology., Danvers, MA).

### Plasmids

The mouse *Angptl8* promoter (−2291/+101 bp) plasmid, which is housed in the region from −2291/+101 bp of *Angptl8* gene, was generated by genomic PCR using sense primer: 5′-CCCAAGCTTTTACGGGATGACTCCCT-3′ and anti-sense primer: 5′-CCCAAGCTTGAGCTGGCTCTGG-3′. Each primer contained a *Hind*3 restriction enzyme site to facilitate subcloning into pGL4.10-Luciferase reporter vector (Promega, Madison, WI). −1508/+101 of fragment was digested from −2291/+101 construct by *Bgl2* restriction enzyme and subcloned. Serially deleted promoter fragments (−966 / +101, −309 / +101 and −59 / +101) were generated by PCR using 5′-GATGGAGAGATCTGGCCTGCTAGC-3′, 5-GAAGATCTGAAGGGAGCCACG-3′ and 5′-GAAGATCTATGGCAGCCTATGG-3′ as sense primers containing a *Bgl*2 restriction enzyme site, respectively and 5′-CCCAAGCTTGAGCTGGCTCTGG-3′ as a common anti-sense primer. Three mutant promoter constructs (−2291/+101) harbouring two different series of mutations or a deletion of the HNF-1-binding site at −84/−68 were generated by GeneArt Site-Directed Mutagenesis System (Invitrogen, Carlsbad, CA). The mutated nucleotide sequences are shown in Fig. 3a. All PCR-generated promoter constructs were verified by sequencing the DNA.

### Cell culture, transfection and luciferase assays

Hepa1–6, HepG2 and HeLa cells were cultured in 12-well plates as previously described. Reporter plasmid (0.3 μg) and 0.5 μg of human HNF-1α and/or HNF-1β expression vector (Addgene, Cambridge, MA) or empty pCMV10 vector (Sigma Aldrich Japan, Tokyo, Japan) were transfected into cells using the Lipofectamine 2000 Transfection Reagent (Invitrogen, Carlsbad, CA). Mouse primary hepatocytes were transfected using GenomeONE-Neo (FD) (Ishihara Sangyo Kaisha, LTD, Osaka, Japan).

### Small interfering (si) RNA against Hnf-1

We purchased siRNA for mouse *Hnf-1α* and *Hnf-1β* (sc-35568 and sc-37929; Santa Cruz Biotechnology, Inc., Santa Cruz, CA). ON-TARGET plus control non-targeting pool (D-001810-10-20, Thermo Fisher Scientific, Lafayette, CO) was purchased to serve as a negative control. The siRNA of each concentrations was transfected into Hepa1–6 cells using Lipofectamine RNAi Max reagent (Invitrogen, Carlsbad, CA). Twenty-four hours after transfection, cells were harvested and whole cell lysates were prepared followed by SDS-PAGE.

### EMSA study

Electrophoretic mobility-shift assays (EMSA, gel-shift assay) were performed as described previously^45,46^. Double-stranded oligonucleotides WT probe, M1 probe and M2 probe were labelled with [*α*-^32^P] deoxy-CTP by a fill-in reaction using a Klenow fragment of DNA polymerase. For competition experiments, a 20- or 40-fold molar excess of cold oligonucleotides was incubated. For supershift experiments, 3 μl of rabbit anti-HNF-1 antibody (sc-8986 ×, Santa Cruz Biotechnology, Inc., Santa Cruz, CA) was added and the mixture was incubated for an additional 30 min at room temperature. All EMSA assays were repeated at least twice with similar results and a representative result is shown.

### Chromatin immunoprecipitation (ChIP) assay

Nuclear extract was prepared from Hepa1–6 cells after incubation with or without 100 nM insulin for 1 hr and subjected to immunoprecipitation. ChIP assays were performed using ChIP-IT Express kit (Active Motif, Carlsbad, CA) according to the manufacturer's protocol. Briefly, formaldehyde (37%) was directly added to the cell culture medium at a final concentration of 1% and incubated for 10 min. Cells were lysed and the nuclear lysate was sonicated three times with 15-sec pulses using a sonicator set at 25% of maximum power to yield the DNA fragments between 150 and 1000 base pairs in length. Sheared chromatin solution was immunoprecipitated with 3 μg of an anti-HNF-1 antibody (sc-8986 ×; Santa Cruz Biotechnology, Inc., Santa Cruz, CA) or preimmune mouse IgG (sc-2025; Santa Cruz Biotechnology, Inc., Santa Cruz, CA). For Ct value-based evaluation, qPCR was performed with power SYBR Green (Applied Biosystems Inc., Foster City, CA) using Applied Biosystems 7500 sequence detector with 1 μl of immunoprecipitate and input samples, according to the manufacturer's specified parameters. The primers for qPCR to amplify the region between −170 and +102 were as follows; sense: 5′-TAGCAGCGTGATATCAGCATTGC-3′ and antisense: 5′-TTGAGCTGGCTCTGGACCACCCAG-3′. The data represent %input ± SEM of tripricate PCR samples. ChIP assays were repeated twice.

### Primary hepatocyte cell culture and insulin stimulation

Hepatocytes were prepared from wild type mice using the collagenase-perfusion method with minor modifications and cultured in Dulbecco’s modified Eagle’s medium (DMEM) containing 10% foetal bovine serum, 0.5 μg/ml insulin, 1 μM dexamethasone, 10 ng/ml epidermal growth factor, 200 μM ascorbic acid, 10 mM nicotine amide, 10 U/ml penicillin and 10μg/ml streptomycin. After a 12 h incubation, cells were cultured in serum- and insulin-free DMEM for 2 hours, and whole-cell lysates were prepared at the time points indicated after treatment with insulin^47^.

### *In-vivo* ChIP assay

*In-vivo* ChIP assyas were performed as previously reported^48^. We employed mouse liver tissue excised from fasted 12 h or re-fed 60 minutes after 12 h fasting mice. Briefly, each sample pf liver tissue (60–80 mg) was weighed and incubated in 1% formaldehyde (1 ml/20 mg tissue) at 37 C for 20 min with agitation. The tissue was then washed twice with ice-cold PBS buffer (PBS/ 1 mM PMSF/ 1 μg/ml aprotinin) and resupended in 100 μl of lysis buffer (1% sodium dodecyl sulfate/ 50 μM Tris-HCl/ 10 mM EDTA/ 1 mM phenylmethylsulphonylfluoride/ 1μg/ml aprotinin) for 10 min at 4 C, The lysate was sonicated three times with 10-sec pulses using a sonicator set at 70% of maximum power to reduce the DNA length to between 200 and 1000 bp. Chromatin solution was used for ChIP assays.

### Statistical analysis

The data were analysed using JMP version 11 (SAS Institute Inc., Cary, NC). The data were analysed using the Dunnett test or Steel method followed by ANOVA. All values are expressed as the mean ± SEM. Significant level was set at p < 0.05

### Equipment and settings

All images of immunoblotting and EMSA study were exposed to photosensitive film (Kodak Japan Ltd, Tokyo) or captured using lumino image analyzer LAS4010 (GE Healthcare Japan, Tokyo). Those images were converted to jpg using Canon TS8000 scanner (Canon Inc, Japan). The images converted jpg was processed to figures using Microsoft PowerPoint (Microsoft corporation, Redmond, WA).

## Supplementary information

Supplementary info.
