# Supplementary material for: Transcriptional Regulation of the Angptl8 Gene by Hepatocyte Nuclear Factor-1 in the Murine Liver
Source: Sci Rep. 2020 Jun 19;10:9999. doi: 10.1038/s41598-020-66570-0 (PMC7305314; doi:10.1038/s41598-020-66570-0)
Supplement: Supplementary file 1 — Supplementary info. [file 41598_2020_66570_MOESM1_ESM.pdf]

Transcriptional Regulation of the *Angptl8* Gene by Hepatocyte Nuclear Factor-1 in the Murine Liver

Takuya Watanabe[1,2], Atsushi Ozawa[1,2*], Shinnosuke Masuda[1], Satoshi Yoshino[1], Emi Ishida[1], Yuri Kondo[1], Shunichi Matsumoto[1], Akiko Katano-Toki[1], Kazuhiko Horiguchi[1], Yasuyo Nakajima[1], Eijiro Yamada[1], Takuya Tomaru[1], Tsugumichi Saito[1], Sumiyasu Ishii[1], Nobuyuki Shibusawa[1], Shuichi Okada[1], Tetsurou Satoh[1] and Masanobu Yamada[1]

1. Department of Internal Medicine, Division of Endocrinology and Metabolism, Gunma University Graduate School of Medicine, 3-39-15 Showa-machi, Maebashi 371-8511, Japan

2. These two authors equally contributed to this work.

**Corresponding author and person to whom reprint requests should be addressed:*

Atsushi Ozawa, MD PhD: E-mail: ozawaa@gunma-u.ac.jp

SUPPLEMENTARY INFORMATION

Supplementary figures: 3

```
-150  ggcctgctta gccatcagtc tgatgcaatg gctgagcctc cgccaggccc

-100  ttgtgcaacc atggccggtt aaccattgac cagggggtc aatggcagcc
                    HNF-1 binding motif
 -50  tatggaaata aaaggcagcc gcagcggccc gggaaccaca cccacgaaac

  +1  TGTCAGCCAT GGCTGTGCTT GCTCTCTGCC TCCTGTGGAC CTTAGCATCA

 +51  GCAGTGCGAC CCGCTCCAGT GGCCCCTCTG GGTGGTCCAG AGCCAGCTCA

+101  ATATGAAGAG CTGACCCTGC TCTTTCACGG GGCCCTGCAG CTAGGCCAGG

+151  CCCTCAATGG CGTGTACAGA GCCACAGAGG CTCGCCTGAC AGAAGCTGGG
```

Supplementary Fig. 1. The nucleotide sequence of the proximal promoter, 5' untranslated (UT) region and exon1 of the murine *Angptl8* gene.

The nucleotide sequences of the proximal promoter, 5' UT region and that of a part of exon 1 are shown in small and large case letters, respectively.

The position of the consensus binding motif for hepatocyte nuclear factor-1 (HNF-1) at -84/-68 is indicated. ATG is indicated in bold font.

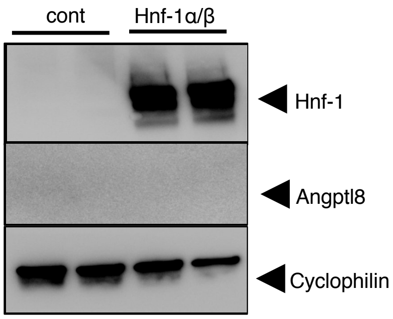

Supplementary Fig. 2. Over-expression of HNF-1α and β did not increased the protein expression level of Angptl8 in non-hepatocyte cells.
After transfection of both HNF-1α and HNF-1β and a control plasmid into HeLa cells, whole cell lysates were prepared and subjected to SDS-PAE.
Immunoblotting was performed using anti-Hnf-1, anti-Angptl8 and anti-cyclophilin antibodies.
The blot of Hnf-1, the blot of Angptl8 and the blot of cyclophilin were cropped from different parts of the same membrane.

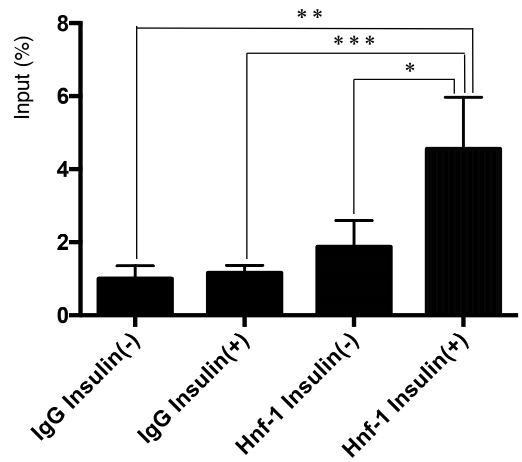

Supplementary Fig. 3. Hnf-1 recruited to the promoter region containing the consensus Hnf-1 binding site at -84/-68 in ChIP assays.

Nuclear extract was prepared from Hepa1-6 cells after incubation with or without 100nM insulin for 1hr and subjected to immunoprecipitation using an anti-HNF-1 antibody or pre-immune IgG (IgG). A ChIP assay was carried out as described in Materials and Methods section. The experiments were performed three times individually, and each PCR was performed in triplicate. The result of these three separate experiments were combined and represent mean ± SEM. An asterisk indicates a significant difference (*$p<0.05$, **$p<0.01$ or ***$p<0.001$).
